# Non-neoplastic indications and outcomes of the proximal and distal femur megaprosthesis: a critical review

**DOI:** 10.1186/s43019-020-00034-7

**Published:** 2020-04-09

**Authors:** Raju Vaishya, Sunil Singh Thapa, Abhishek Vaish

**Affiliations:** Department of Orthopaedics and Joint Replacement Surgery, Indraprastha Apollo Hospital, SaritaVihar, New Delhi, 110076 India

**Keywords:** Megaprosthesis, Total knee arthroplasty, Total Hip Arthroplasty, Bone tumors, Neoplasia, Fractures

## Abstract

**Purpose:**

Megaprosthesis or endoprosthetic replacement of the proximal and distal femur is a well-established modality for treatment of tumors. The indications for megaprosthesis have been expanded to the treatment of some non-neoplastic conditions of the knee and hip, with the severe bone loss associated with failed arthroplasty, communited fractures in the elderly with poor bone quality, and resistant non-union. Th aim of this study is to find out whether megaprosthesis of the knee and hip is successful in the treatment of non-neoplastic condtions. The study comprises a review of the indications, complications, and outcomes of megaprosthesis of the proximal and distal femur in non-neoplastic conditions of the knee and hip joints.

**Methods:**

We extensively reviewed the literature on non-neoplastic indications for megaprosthesis of the proximal and distal femur after performing a detailed search of the Pubmed database using the medical subject heading (MeSH) terms ‘proximal femur replacement’ or ‘distal femur replacement’ and ‘hip or knee megaprosthesis.’ The data obtained after the structured search were entered into a Microsoft Excel spreadsheet. The frequency distribution of the demographic data, indications, complications, and outcome was calculated.

**Result:**

We included ten studies (seven proximal femur replacement and three distal femur replacement) of 245 proximal femur and 54 distal femur mega prostheses for treatment of non-neoplastic conditions. Bone loss in failed arthroplasty, either due to periprosthetic fracture or deep infection, was the most common indication for megaprosthesis. Dislocation was the most common complication after proximal femur megaprosthesis, and infection was the leading cause of complications after distal femur megaprosthesis.

**Conclusion:**

Megaprosthesis for treatment of non-neoplastic conditions around the distal and proximal femur is a viable option for limb salvage, with an acceptable long-term outcome. Although the complications and survival rates of megaprosthesis in non-neoplastic conditions are inferior to a primary arthroplasty of the hip and knee but are comparable or better than the mega prosthetic replacement in the neoplastic conditions. Proximal femoral megaprosthesis has higher dislocation rates and requirement for revision compared to distal femoral megaprosthesis. However, the proximal femoral megaprosthesis has lower rates of infection, periprosthetic fractures, and soft tissue complications, as compared to distal femoral megaprosthetic replacement. Both associated with aseptic loosening but not statistically significant.

## Summary

Megaprosthesis or endoprosthetic replacement of the proximal and distal femur is a well-established modality for the treatment of tumors. The indications for megaprosthesis have been expanded to the treatment of some non-neoplastic conditions of the knee and hip, with the severe bone loss associated with failed arthroplasty, communited fractures in the elderly with poor bone quality, and resistant non-union. Very few systematic reviews are available on proximal or distal femoral replacement for treatment of non-neoplastic conditions. This study reviews the indications, complications, and the outcomes of the megaprosthesis of the proximal and distal femur, in non-neoplastic conditions of the knee and hip joints. We included ten studies (seven on proximal femur replacement and three on distal femur replacement) of 245 proximal femur and 54 distal femur megaprostheses for treatment of non-neoplastic conditions. Bone loss in failed arthroplasty, either due to periprosthetic fracture or deep infection was the most common indication for megaprosthesis. Dislocation was the most common complication after proximal femur megaprosthesis and infection was the leading cause of complications after distal femur megaprosthesis. Proximal and distal femur megaprosthesis can be used as a salvage procedure in non-neoplastic conditions, with massive bone loss. Megaprosthesis for treatment of non-neoplastic conditions around the distal and proximal femur is a viable option for limb salvage, with an acceptable long-term outcome. Although the complications and survival rates after megaprosthesis in non-neoplastic conditions are inferior to primary arthroplasty of the hip and knee but are comparable or better than the mega prosthetic replacement in the neoplastic conditions. Proximal femoral megaprosthesis has higher dislocation rates and requirement for revision compared to distal femoral megaprosthesis. However, proximal femoral megaprosthesis has lower rates of infection, periprosthetic fractures, and soft tissue complications, as compared to distal femoral megaprosthetic replacement. Both of these procedures have a statistically insignificant difference in the aseptic loosening of the prosthesis. Dislocations in proximal femur megaprosthesis and infection in distal femur megaprosthesis are the major significant complications.

## Introduction

Megaprosthesis or endoprosthetic replacement has been the standard of care for orthopaedic oncology for many decades [11]. Severe bone stock deficiency in the proximal or distal femur, as seen in septic or aseptic failed hip or knee arthroplasty and osteoporotic fracture in the elderly with severe comminution or failed fracture fixation, precludes the use of conventional prostheses. The treatment options available in such a situation are structural allograft-prosthesis composite, impaction allografting, long cemented or press-fit revision stem resection arthroplasty and megaprosthesis [1, 21]. There are many limitations associated with the use of allograft for reconstruction in bone loss, thus increasing the use of megaprosthesis for tumor surgery [5, 6]. Encouraging results of the successful outcome of megaprosthesis for tumor salvage in the proximal and distal femur have broadened the indications for megaprosthesis for the treatment of non-neoplastic conditions with extensive bone loss in the proximal or distal femora [8, 19].

Very few systematic reviews are available on proximal or distal femoral replacement for treatment of non-neoplastic conditions [20, 24]. Two recent systematic reviews on megaprosthesis for treatment of non-neoplastic conditions of the proximal and distal femur found overall midterm survival rates of 76% and 83% for proximal and distal femoral prostheses, respectively [14, 15]. The main aim of this study is to review the literature and analyze the demography, indications, complications, and outcomes of proximal or distal femur megaprosthesis for the treatment of non-neoplastic conditions. We also attempted to compare the complications and outcomes of proximal and distal femoral megaprosthesis.

## Material and methods

### Literature search

We searched the Pubmed database for literature on megaprosthesis of the proximal or distal femur for the treatment of non-neoplastic conditions, to access the most relevant studies, on 10 July 2019. The keywords used in the Pubmed search were ‘proximal femoral replacement’ or ‘distal femur replacement’ and ‘hip or knee megaprosthesis.’

### Eligibility criteria

The inclusion criterion was articles that described the use of a proximal or distal femur megaprosthesis for treatment of non-neoplastic conditions. Case reports and the reports on the use of megaprosthesis for treatment of tumors were excluded.

### Data collection

The authors screened the abstracts of possibly relevant articles and studied the full text of those articles meeting the inclusion criterion. Articles on proximal femur and distal femur megaprosthesis were reviewed separately. Data were extracted on type of study, number of patients, age, indications, complications, and follow up. Complications were classified according to the system reported by Henderson et al. [12], as previously modified for use in non-neoplastic conditions [14], as soft-tissue complications (type 1), aseptic loosening (type 2), structural complications or periprosthetic fracture (type 3), and peri-megaprosthetic infections (type 4). Data on revision and survival rates were also recorded when available. The data were then registered in a Microsoft Excel sheet, and the frequency distribution and the mean were calculated.

## Results

### Search results

**Fig. 1:**
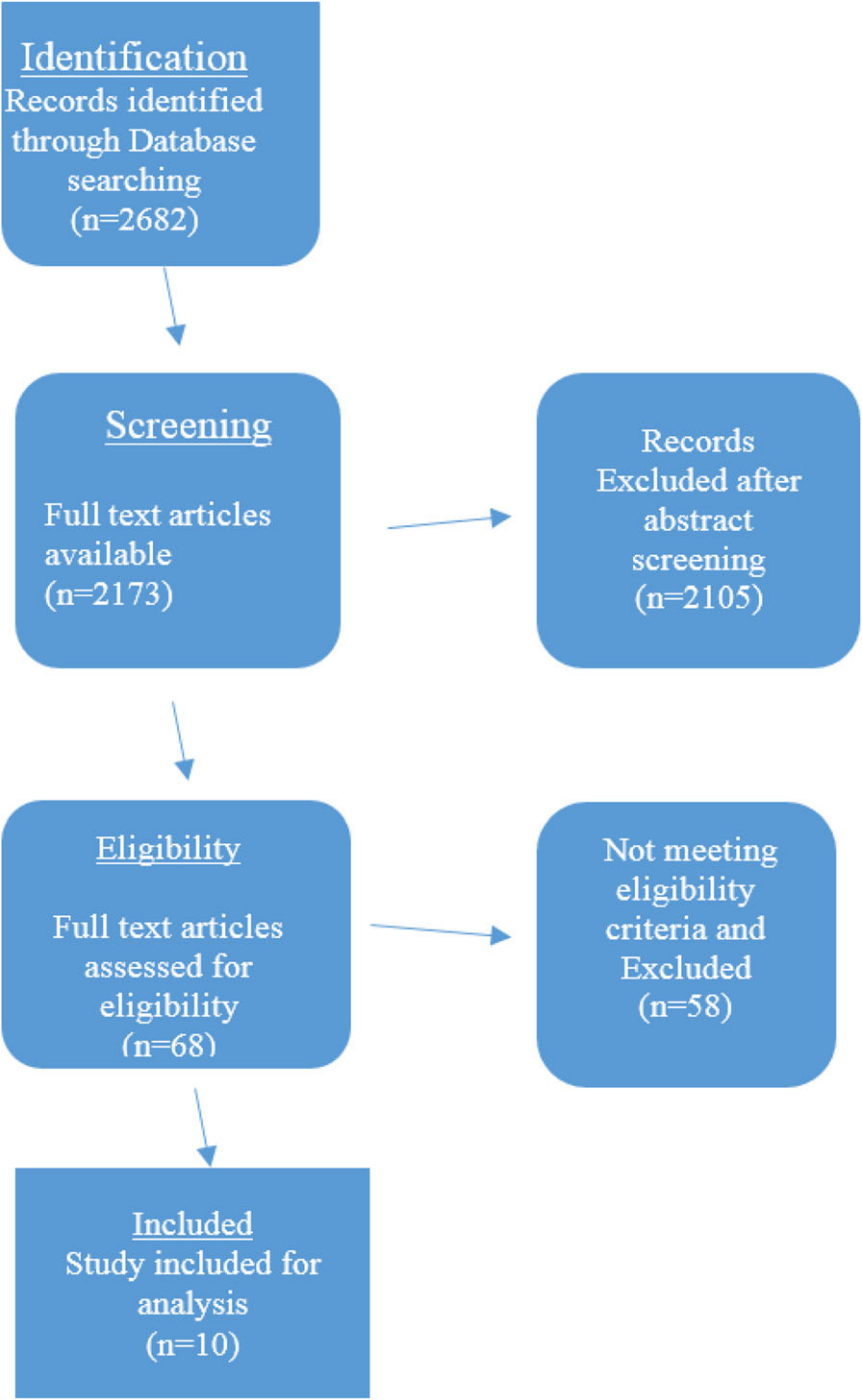
Flow chart illustrating search strategy

A total of 2682 articles was identified after the initial Pubmed search. 2173 articles revealed full available texts which were further screened. All the relevant articles on non-neoplastic conditions were then screened. All the articles with full text and those meeting the inclusion criterion were selected for this study. A total of ten studies (seven on proximal femur megaprosthesis and three on distal femur megaprosthesis) fulfilled the eligibility criterion. The search strategy is illustrated in Fig. 1.

Out of ten studies, four on proximal femur megaprosthesis and two on distal femur megaprosthesis were prospective studies. We analyzed data on 245 proximal femur megaprostheses (in 243 patients) and 54 distal femur megaprostheses (in 54 patients). These studies had a sample size ranging from 8 to 79 (Tables 1 and 3).

**Table 1:** Study design, number of prostheses, mean age, and follow up of proximal femur megaprosthesis

Authors	Study design	Number of cases of megaprosthesis	Age, years	Follow up, months
Malkani AL et al.; 1995 [20]	Retrospective	32 (33 prostheses)	60.6	133.2
Parvizi J et al.; 2007 [3]	Retrospective	48	73.8	36.5
Shih ST et al.; 2007 [22]	Prospective	12	59 (25–75)	68.4 (3.3–9 years)
Dean BJ et al.; 2012 [23]	Prospective	8	67.5 (50–79)	16.5 (6–36)
McLean AL et al.; 2012 [24]	Prospective	20	72 (36–91)	48 (12–116)
Grammatopoulos G et al.; 2016 [25]	Retrospective	79 (80 prostheses)	69 (28–93)	60 (0–11.5 years)
Viste A et al.; 2017 [26]	Prospective	44	79 (53–97)	72 (2–12 years)
Total		243 (245 prostheses)	68.7 years	44.64

#### Proximal femur megaprosthesis (*n* = 245)

The mean age of the patients was 68.7 years, and the mean follow-up duration was 44.64 months (Table 1). The indications for surgery were periprosthetic infection (28.9%), periprosthetic fracture (28.1%), and massive bone loss due to arthroplasty and complex fractures or failed internal fixation (22.8%) (Table 2). Dislocation of the prosthesis was the most common complication (14.6%, *n* = 32), followed by periprosthetic fracture and aseptic loosening in 7.5% and 6.9% of cases, respectively (Table 2). The Harris hip score improved from a preoperative mean of 38.9 to a mean of 72.6 at the last follow up. Out of seven study reports, only four [3, 20, 25, 26] discussed revision and implant survival. A revision was required in 32 cases,and the mean implant survival was 80% at 5 years (Table 2).

**Table 2:** Indications, complications, Harris hip score, and revision and implant survival of proximal femur megaprosthesis

Authors	Indications	Complications	Harris Hip Score	Revision surgery	Implant survival
Malkani AL et al.; 1995 [20]	Massive femoral bone loss (*n* = 33)	Dislocation (*n* = 11) Aseptic loosening (*n* = 11)	Preop 46 (31–83) Postop 80 (50–91)	Revision (*n* = 11)	64% at 12 years
Parvizi J et al.; 2007 [3]	Periprosthetic fracture (*n* = 20) Infection (*n* = 13) Failed arthroplasty (*n* = 13) Non-union of fracture (*n* = 1) Radiation AVN (*n* = 1)	Dislocation (*n* = 8) Aseptic loosening (*n* = 4) Periprosthetic infection (*n* = 1)	Preop 37.1 (15–61) Postop 64.9 (13–91)	Revision (*n* = 10)	87% at 1 year and 73% at 5 years
Shih ST et al.; 2007 [22]	Periprosthetic fracture (*n* = 2) Periprosthetic infection (*n* = 6) Aseptic loosening (*n* = 3) Allograft fracture (*n* = 1)	Dislocation (*n* = 5) Aseptic loosening (*n* = 1) Displaced fracture (*n* = 3) Deep infection (*n* = 4) Leg shortening (*n* = 2) Ectopic ossification (*n* = 1)	Preop 30 (16–42) Postop 83 (68–92)	Not mentioned	Not mentioned
Dean BJ et al.; 2012 [23]	Failed internal fixation for proximal femur fracture (*n* = 8)	No	71.4 (64–85)	Not mentioned	Not mentioned
McLean AL et al.; 2012 [24]	Periprosthetic femoral fracture (*n* = 20)	Dislocation (*n* = 3) Periprosthetic fracture (*n* = 1)	Not mentioned	Not mentioned	Not mentioned
Grammatopoulos G et al.; 2016 [25]	Periprosthetic joint infection (*n* = 40) Periprosthetic fracture (*n* = 12) Failed osteosynthesis of a fracture (*n* = 16) Miscellaneous (*n* = 9)	Infection (*n* = 9) Dislocation (*n* = 3).	NA	revision (*n* = 9)	87% (95% CI 76– 98%) at 5 years
Viste A et al.; 2017 [26]	Aseptic loosening (*n* = 16) Periprosthetic fracture (*n* = 15) Prosthetic joint infection (*n* = 12) Instability (*n* = 1)	Dislocation (*n* = 6) Aseptic loosening(n = 1) Periprosthetic fracture (*n* = 4) Infection (*n* = 1)	Preop 42.8 (25.9–82.9) Postop 68.5 (21.0–87.7)	Revision (*n* = 2)	86% at 5 years and 66% at 10 years
Total	1. Periprosthetic fracture (*n* = 69, 28.1%) 2. Periprosthetic infection (*n* = 71, 28.9%) 3. Massive femoral bone loss in arthroplasty (*n* = 33, 13.4%) 4. Failed internal fixation for proximal femur fracture and complex fracture (*n* = 24, 9.7%) 5. Aseptic loosening (*n* = 19, 7.7%) 6. Miscellaneous (*n* = 16, 6.5%) 7. Failed arthroplasty (*n* = 13, 5.3%)	1. Dislocation (*n* = 36, 14.6%). 2. Aspetic loosening (*n* = 19, 7.5%) 3. Periposthetic fracture (*n* = 8, 3.2%) 4. Periposthetic infection (*n* = 17, 6.9%). *miscallenous soft tissue complications (n = 3)	Mean preop = 38.9 and mean post op = 72.6	Revision (*n* = 32, 13.06%)	Mean − 80% at 5 years

*AVN* avascular necrosis, *Preop* preoperative, *Postop* postoperative

#### Distal femur megaprosthesis (*n* = 54)

The mean age of the patients was 75.49 years, and the mean follow-up duration was 43.05 months (Table 3). The most common indication for distal femur megaprosthesis was substantial bone loss after failed knee arthroplasty in 55.5% of cases (Tables 2 and 4). Periprosthetic infection was the most common complication (18.5% cases) (Tables 2 and 4).

**Table 3:** Study design, number of prostheses, mean age, and follow up of distal femur megaproshthesis

Authors	Study design	Number of patients	Age (years)	Follow up (months)
Vaishya R et al.; 2011 [12]	Prospective	10	74.38 (± 10.1)	48
Fakler JK et al.; 2013 [27]	Prospective	14	77	27
Vertesich K et al.; 2019 [28]	Retrospective	30	75.1	54.15 (1–240)
Total		54	75.49	43.05

**Table 4:** Indications, complications, Knee Society Score, and revision and implant survival of distal femur megaprosthesis

Authors	Indications	Complications	Knee Society Score (KSS)	Outcomes	Implant survival
Vaishya R et al.; 2011 [19]	Resistant non-union supracondylar fracture (*n* = 10)	Wound necrosis (*n* = 2) Periprosthetic fracture (*n* = 1)	Not mentioned	Not mentioned	Not mentioned
Fakler JK et al.; 2013 [27]	Periprosthetic fracture (*n* = 6) Complex fractures and extensive bone defects (*n* = 8)	Patellar tendon rupture (*n* = 1) Early aseptic loosening (*n* = 1) Periprosthetic frature (*n* = 4) Infection (*n* = 2)	KSS improved significantly from 20.0 (IQB 7.5–30.0) points preoperatively to 80.0 (IQB 62.3–89.0)	Not mentioned	Not mentioned
Vertesich Ket al; 2019 [28]	Substantial bone loss following TKA (*n* = 30)	Soft-tissue complications (*n* = 3) Aseptic loosening (*n* = 4) Periprosthetic fracture (*n* = 1) Infection (*n* = 8)	DFR achieved 69.3% of KSS pain score, 23.1% KSS function score and 76.2% of ROM compared to patients with primary TKA	Revision surgery (*n* = 3) Transfemoral amputation (*n* = 4) Distal femur reconstruction (*n* = 1)	Revision-free survival was 74.8% at 1 year, 62.5% at 3 years and 40.9% at 10 years postop
Total	Substantial bone loss after failed TKA (*n* = 30) Resistant nonunion supracondylar fracture (*n* = 10) Traumatic fracture with severe comminution (*n* = 8) Periprosthetic fracture (*n* = 6)	Soft tissue complications (*n* = 6) Aseptic loosening (*n* = 5) Periprosthetic fracture (*n* = 6) Periposthetic infection (*n* = 10)		Revision surgery (*n* = 3, 5.5%) Transfemoral amputation (*n* = 4) Distal femur reconstruction (*n* = 1)	

*TKA* total knee arthroplasty, *N*A not available, *ROM* range of motion, *preop* preoperative

The Knee Society Score improved from a preoperative median of 20 to a postoperative median of 80 [27]. Out of three studies, only Vertesich et al. [27] mentioned revision and implant survival in their study. Revision was required in three cases, and the implant survival was 74.8% at 1 year and 40.9% at 10 years (Table 4).

### Comparison of complications of the proximal femur and distal femur megaprosthesis

**Fig. 2:**
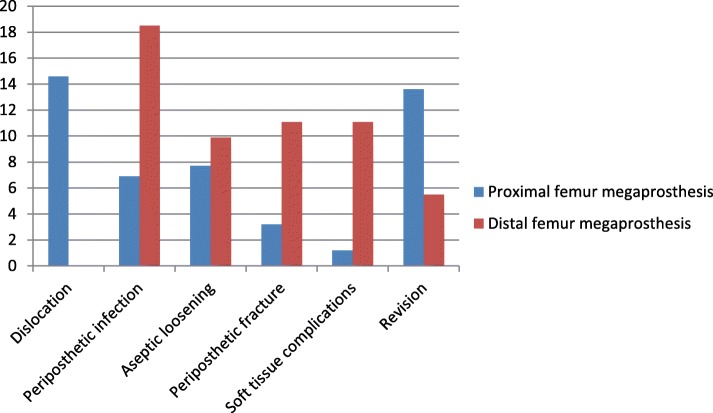
Comparison of complications of proximal versus distal femur megaprosthesis (expressed in percentage)

Proximal femoral megaprosthesis has higher dislocation rates and requirement for revision compared to distal femoral megaprosthesis. However, proximal femoral megaprosthesis is associated with lower rates of infection, periprosthetic fractures, and soft tissue complications, as compared to distal femoral megaprosthetic replacement. Both of these procedures have a statistically insignificant difference in the aseptic loosening of the prosthesis (Fig. 2).

## Discussion

Megaprosthesis of the proximal or distal femur is a viable option for limb reconstruction in non-neoplastic conditions like failed hip or knee arthroplasty, periprosthetic fractures, osteoporotic fracture with severe comminution, or resistant non-union in elderly patients [2, 29]. Megaprosthesis in such cases should be considered as a limb salvage option in carefully selected patients when other surgical options are not feasible [14]. In this review, we analyzed the complications and outcome of proximal and distal femur megaprosthesis.

Failed hip arthroplasty with extensive bone loss (due to infection, fracture, or aseptic loosening) was the most common (83.6%) non-neoplastic indication for proximal femur megaprosthesis (Table 2). Failed total knee arthroplasty (55.5%) was the most common non-neoplastic indication for distal femur megaprosthesis (Table 4).

Dislocation of the hip prosthesis is the most common complication observed in this review, seen in 14.6% of proximal femur megaprostheses (but in none of the distal femur megaprostheses: Table 4). Our results are in agreement with the systematic review by Korim et al. [14], reporting a 15.7% rate of hip dislocation at a mean follow up of 45 months. The cause of instability is multifactorial, including inability to achieve a secure repair of the residual soft tissues around the metal prosthesis [9] and compromised abductors around the hip due to multiple previous reconstructive procedures. The monobloc implants used previously were less versatile and often led to dislocation, but with the new generation of megaprosthesis providing better provision for more secure soft tissue reattachment and the ability to reapproximate the retained proximal host bone to the prosthesis, the rate of dislocation has decreased [21].

Periprosthetic infection was seen in 6.9% of proximal femur megaprostheses (hip) and in 18.5% of distal femur megaprostheses (knee), thus agreeing with previous findings of a mean rate of 7.6% for proximal femoral prostheses [13, 14] and 15% for distal femoral prostheses [15]. A recent systematic review has reported a mean rate of peri-megaprosthetic infection of 10%, following tumor resection [22]. The overall infection rate in hip and knee arthroplasty is as low as 1% [16, 17]. Periprosthetic infection is common and remains the most challenging complication after megaprosthesis because of poor-quality soft tissue due to multiple previous surgeries, poor overall health status, and long operating times [16, 18, 22]. These factors result in a poor functional outcome and failed limb salvage.

Aseptic loosening was seen in 7.7% and 9.9% of proximal and distal femur megaprosthesis, respectively. Aseptic loosening of megaprosthesis in the treatment of non-neoplastic diseases has been previously detected with rates ranging from 0% to 9.5% [25, 29], and these reports are consistent with aseptic loosening after tumor prosthesis [4]. Periprosthetic fracture was seen in 3.2% and 11.1% of cases of proximal and distal femur megaprosthesis, respectively. The mean age of the distal femur cohort was 75.49 years compared to 68.7 years in the proximal femur group; poorer bone quality may be the reason for a higher rate of periprosthetic fracture in this group [26].

Soft tissue complications were seen in 1.2% and 11.1% of cases of proximal and distal femur megaprosthesis, respectively (Table 4). In a retrospective review of 2174 patients, Henderson et al. [12] detected an overall rate of soft-tissue complications (i.e., including dislocation) of 5.2% with primary proximal femoral prosthesis. We found that revision was required in 13.06% and 5.5% cases of proximal and distal femur megaprosthesis, respectively. In a systemic review by Korim [9], reoperation rates ranged from 13.3% to 40% in proximal femur megaprosthesis.

We found significant improvement in the Harris hip score after proximal femur megaprosthesis and significant improvement in the Knee Society Score after distal femur megaprosthesis. We found that the mean 5-year survival of proximal femur megaprosthesis was 80%, which is comparable (78–90%) to that reported for neoplastic indications [7, 8, 19].

The main limitations of this review were the heterogeneity and the small sample size of the study. Data on the patients who were lost to follow up in most of the studies was lost. Details of the complications and their outcomes could not be assessed thoroughly, as none of the articles except for that of Grammatopoulos et al. [10] reported the complications for each indication and we were able to analyze only three studies on distal femur megaprosthesis.

## Conclusion

Proximal and distal femur megaprosthesis can be used as a salvage procedure for non-neoplastic conditions, with massive bone loss. Proximal femoral megaprosthesis has higher dislocation rates and requirement for revision compared to distal femoral megaprosthesis. However, the proximal femoral megaprosthesis is associated with rates of infection, periprosthetic fractures, and soft tissue complications, as compared to distal femoral megaprosthetic replacement. Both of these procedures have a statistically insignificant difference in the aseptic loosening of the prosthesis. Dislocations in proximal femur megaprosthesis and infection in distal femur megaprosthesis are the major significant complications.

## Data Availability

Patient-related data were collected from the department with permission of the patient and the treating consultant (author, Dr Raju Vaishya).
